# Hospital Methods at Melbourne

**Published:** 1907-08-31

**Authors:** 


### HOSPITAL METHODS AT MELBOURNE.

To judge from an article which appeared in the *Melbourne Argus* of June 13, the methods adopted in the election of the medical and surgical staff of the Melbourne Hospital have special and peculiar qualities which may well claim attention. Whether they will also be deemed worthy of admiration is, however, another question. It appears that the honorary appointments to the staff are made for a term of four years, and that the holders of these appointments are eligible for re-election. But such re-election is by no means a formal and foregone conclusion. On the contrary, when the vacancies are declared, there is an entirely open field, and an active competition is promptly set in progress. The sole advantage extended to those candidates who present themselves for re-election is that the name of each such candidate appears on the list of applicants with an asterisk attached to it. Now we are very far from saying that the holders of hospital appointments may not at regular intervals be profitably subject to review. Hospital physicians and surgeons, like other mortals, are all the better for influences which remind them that their own personal interests and positions may be imperilled by an imperfect attention to official claims and responsibilities. Hence, provided the reviewing authorities are informed and impartial, we can see no objection to an arrangement which brings the work and efficiency of the several members of a hospital staff under periodical scrutiny. Whether the Melbourne methods satisfy these conditions our readers will judge when we have described them.

The commanding facts in the election are two in number. First, all the governors of the hospital are electors. Secondly, every person who pays £1 to the funds of the hospital becomes a governor for the year within which such payment is made. The inevitable results of such an arrangement are obvious. When an election approaches it is found that the number of the subscribers to the hospital funds becomes greatly increased. The personal friends of each of the candidates become in this way voters, with an opportunity to make their influence felt at the poll. From this, naturally, emerges all the recognised machinery necessary to conduct what is practically a popular election. "Pocket governors" are created by scores or even hundreds. Rival committees conduct an active canvass for their several candidates. Election literature of the usual perfervid character makes its appearance. Vehicles to convey voters to the poll are chartered. And personal, sectarian, and numerous other forms of influence are set into active operation. Of course, all this costs money. In one such contest it is said a candidate who was defeated spent not less than £2,000, and it is exceptional for an applicant to escape with an outlay of less than £300; even those who seek re-election may be put to very considerable expense. Indeed, it has hitherto been the custom for the retiring staff to contribute each £50 or £100 to a common fund, and in this way to add some hundreds of "governors" for the purpose of the election, and each pledged to vote the "ticket."

We imagine that few persons who have any experience of hospital management will sympathise with the proceedings just described, and members of the medical profession must surely view them with profound dislike. In the first place, it is obvious that such a method of appointment must lead to the substitution of personal, or even of less desirable, considerations, for those which are really of importance —namely, professional efficiency and value. Again, the power of the purse is introduced into a contest where its note should never be heard. It is inevitable, too, that amidst an election so conducted professional reserve and dignity must frequently be seriously qualified. A competition downwards sets in, and *facilis descensus Averno*. More important than all, is it not obvious, that, in the circumstances described, the real and essential issues, that is, the efficient care of the patients, the successful conduct of clinical instruction, and the advance of professional knowledge, are apt only too readily to be altogether forgotten. We say nothing here of the practice of throwing hospital appointments open every few years to unguarded competition, though such a course has grave disadvantages. But we do most strongly deprecate a custom which commits the responsible choice of hospital physicians and surgeons to the tumult of a popular election in a manufactured and irresponsible constituency.

